# Mediterranean lifestyle and risk of adverse cardiovascular events, all-cause and cardiovascular mortality among individuals with type 2 diabetes

**DOI:** 10.1016/j.eclinm.2026.104134

**Published:** 2026-08-07

**Authors:** María del Carmen Aznar de la Riera, Jesús Díaz-Gutiérrez, Stefanos N. Kales, Miguel Ruiz-Canela, Almudena Castro Conde, Beth Frates, Fernando Rodríguez-Artalejo, Mercedes Sotos-Prieto

**Affiliations:** a Department of Preventive Medicine and Public Health, School of Medicine, Universidad Autónoma de Madrid, Calle del Arzobispo Morcillo, 4, 28029, Madrid, Spain; b CIBERESP (CIBER of Epidemiology and Public Health), Av. Monforte de Lemos, 3-5, 28029, Madrid, Spain; c Sant Pau Research Institute (IR SANT PAU), Barcelona, Spain; d Department of Cardiology, Hospital de la Santa Creu i Sant Pau, Barcelona, Spain; e Department of Environmental Health, Harvard T.H. Chan School of Public Health, 665 Huntington Avenue, Boston, MS, 02115, USA; f Occupational Medicine, The Cambridge Health Alliance/ Harvard Medical School, USA; g Biomedical Research Center Network on Physiopathology of Obesity and Nutrition (CIBEROBN), Institute of Health Carlos III, Madrid, Spain; h University of Navarra, Department of Preventive Medicine and Public Health, Institute of Nutrition and Health, Pamplona, Navarra, Spain; i IdiSNA, Navarra Institute for Health Research, Pamplona, Spain; j Department of Cardiology, University Hospital La Paz, IdiPAZ, Biomedical Research Center-Cardiovascular Diseases (CIBERCV-ISCIII), 28046, Madrid, Spain; k Department of Physical Medicine and Rehabilitation, Harvard Medical School, Spaulding Rehabilitation Hospital Charlestown, MA, USA; l IMDEA-NUTRITION Institute. CEI UAM+CSIC, Ctra. de Canto Blanco 8, E. 28049, Madrid, Spain

**Keywords:** Type 2 diabetes, Mediterranean diet, Mediterranean lifestyle, Cardiovascular disease, Secondary prevention, Epidemiology

## Abstract

**Background:**

Individuals with type 2 diabetes experience a significantly increased risk of major adverse cardiovascular events (MACE), highlighting the need for effective management tools. Although the Mediterranean Lifestyle has been associated with a lower risk of cardiovascular disease (CVD) in the general population, evidence among adults with type 2 diabetes remains limited. The aim of this study was to evaluate the association between adherence to the Mediterranean Lifestyle Index (MEDLIFE) and risk of MACE, all-cause and CVD mortality in British adults with type 2 diabetes.

**Methods:**

We analyzed data from 3612 individuals aged 40–70 from the UK Biobank cohort, who had type 2 diabetes at baseline (2009–2012) and were followed up until 2024. The MEDLIFE consisted of 25 items, organized into three blocks: (i) Mediterranean food consumption, (ii) Mediterranean dietary habits, and (iii) Physical activity, rest, social habits and conviviality (range 0–25, highest adherence). Incident MACE data were obtained through hospital records, while mortality data were ascertained from the National Death Index. Cox regression models were used to estimate hazard ratios (HR) and 95% confidence intervals (CI) for the outcomes studied, adjusting for potential confounders.

**Findings:**

After a median follow-up of 10.5–12.1 years, 737 (20.4%) MACE, 171 (4.73%) CVD deaths, and 645 (17.9%) all-cause deaths were identified. Higher adherence to the MEDLIFE was associated with stepwise decreases in MACE risk, all-cause and CVD mortality. Compared to participants in the lowest MEDLIFE quartile, the HR (95% CI) for those in the highest quartile was 0.73 (0.59–0.91) for MACE, 0.74 (0.58–0.93) for all-cause mortality, and 0.49 (0.30–0.81) for CVD mortality (all p-trend <0.01). Each MEDLIFE block contributed to the observed associations. Analyses remained robust throughout sensitivity analyses.

**Interpretation:**

Higher adherence to the MEDLIFE was associated with significantly lower risks of MACE, all-cause and CVD mortality among British individuals with type 2 diabetes. Our findings support the MEDLIFE as a promising and accessible tool for type 2 diabetes management.

**Funding:**

This work was supported by the 10.13039/501100004587Carlos III Health Institute, the Secretary of R+D+I; the 10.13039/501100008530European Regional Development Fund/10.13039/501100004895European Social Fund (FIS grants 22/1111; 23/00079); National Agency of Research (CNS2022-135623); Ministerio de Educación, Formación Profesional y Deportes (FPU contract to MDCAR).

## Graphical abstract

**Figure:**
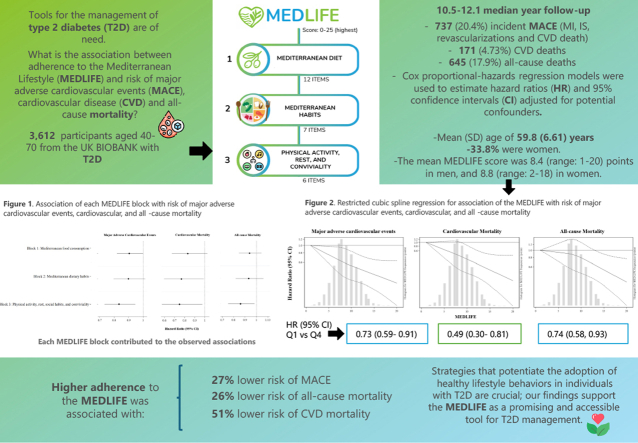

**Research in context**

**Evidence before this study**

Individuals with type 2 diabetes face an increased risk of major adverse cardiovascular events (MACE), highlighting the need for effective management tools. We searched PubMed for articles published from database inception to April 1st, 2026, with no language restrictions, using the search terms “Mediterranean lifestyle”, “type 2 diabetes”, “Major Adverse Cardiovascular Events”, and “Secondary Prevention” and further hand-searched reference lists of relevant articles. Although the Mediterranean Lifestyle Index (MEDLIFE) has been associated with lower risk of cardiovascular disease (CVD) in the general population, evidence among adults with type 2 diabetes remains limited, especially beyond the Mediterranean Basin.

**Added value of this study**

To our knowledge, this is the first study to evaluate the association between the MEDLIFE and risk of major adverse cardiovascular events (MACE), all-cause and CVD mortality in British individuals with type 2 diabetes. The MEDLIFE was associated with a substantially lower risk of MACE, all-cause and CVD mortality. Each MEDLIFE block (Mediterranean food consumption, Mediterranean dietary habits, and Physical activity, rest, social habits and conviviality) contributed to the observed associations, underscoring the importance of a comprehensive healthy lifestyle.

**Implications of all the available evidence**

Our findings support the MEDLIFE as a promising and accessible tool for type 2 diabetes management.

## Introduction

The global prevalence of type 2 diabetes is expected to increase by nearly 62% between 2021 and 2050, affecting more than 1.27 billion people.^1^ Cardiovascular disease (CVD) is the primary complication, and the risk of major adverse cardiovascular events (MACE), including myocardial infarction (MI), ischemic stroke (IS), heart failure (HF), revascularization procedures, and CVD death, may be up to 2.5-fold higher in individuals with type 2 diabetes.^2^ Moreover, more than one-third of the diabetes related deaths occur in people under the age of 60, highlighting the need for public health interventions.^3^

Although comprehensive strategies for CVD risk reduction, such as lifestyle counseling and advances in glucose and lipid control have emerged in recent years, the risk for MACE in patients with type 2 diabetes remains high.^4^ Individuals with an early-onset of type 2 diabetes show more obesity, dyslipidemia, tobacco smoking, sedentary lifestyle and a diminished glucose control, all of which contribute to a poor prognosis.^5^ In turn, adherence to a healthy dietary pattern, such as the Mediterranean Diet (MedDiet) may lower the risk for MACE and CVD mortality in individuals with type 2 diabetes, as observed in the Prevención con Dieta Mediterránea (PREDIMED) trial and further recommended by the American Diabetes Association (ADA).^4^^,^^6^^,^^7^ Regular physical activity,^8^ optimal social connection,^9^ and adequate sleep^10^ have also shown to lower the risk of MACE and mortality among individuals with type 2 diabetes.^11^ Consequently, several cohort studies have evaluated multi-component combinations of the aforementioned lifestyles, and their findings have shown a lower risk for multiple adverse outcomes, such as CVD^12^ and all-cause and cause-specific mortality.^13^

However, less is known about the combination of the traditional lifestyle practices characteristic of the populations residing in the Mediterranean Basin with respect to the risk of MACE. In this context, the MEDiterranean LIFEstyle index (MEDLIFE) is a validated tool that assesses compliance with a Mediterranean lifestyle. Higher scores in the MEDLIFE have been associated with lower risk of CVD,^14^ metabolic syndrome^15^ and mortality^15^ in Mediterranean countries. Moreover, the MEDLIFE has been associated with lower risk of MACE^16^ and reversion of metabolic syndrome^17^ in patients with established CVD. Outside the Mediterranean region, higher adherence to the MEDLIFE has also been associated with lower risks of type 2 diabetes,^18^ and microvascular complications among individuals with prediabetes^19^; however, evidence remains scarce. Thus, despite its established associations with non-communicable diseases (NCD), evidence on the potential benefits of the MEDLIFE in individuals with type 2 diabetes—particularly in non-Mediterranean populations—remains limited. This is important because developing and implementing cost-effective strategies that may have long-lasting health impact in individuals with type 2 diabetes remains a persistent challenge.^4^

Accordingly, we aimed to evaluate the association between adherence to the MEDLIFE and risk of MACE, all-cause and CVD mortality in a middle-aged, non-Mediterranean population with type 2 diabetes. Furthermore, we investigated the potential modification by factors related to diabetes prognosis, such as glycemic control, diabetes duration and type 2 diabetes medication use.

## Methods

### Study design and population

The UK Biobank is a multicenter prospective cohort study with over half a million participants aged 40–69, recruited between 2006 and 2010 in Wales, Scotland, and mostly in England. Detailed information about this cohort has been previously reported.^20^ Briefly, participants provided information on socio-demographic characteristics and lifestyle factors through a touch-screen questionnaire and a computer-assisted interview. They also underwent a physical examination, and biological samples were collected following standardized procedures. In addition, they completed up to five online 24 h-dietary assessments using the validated Oxford WebQ^21^^,^^22^ questionnaire every 3–4 months from 2009 to 2012 (cycle 0: April 2009 to September 2010; cycle 1: February 2011 to April 2011; cycle 2: June 2011 to September 2011; cycle 3: October 2011 to December 2011; and cycle 4: April 2012 to June 2012). For the present analysis, we used data from 5970 participants who had completed at least two dietary assessments and had type 2 diabetes at baseline. In line with previous studies, diagnosed participants were identified through self-reporting, general practice, or hospital records of type 2 diabetes diagnosis.^13^ Undiagnosed participants at baseline were identified, according to the ADA criteria, based on random glucose level ≥11.1 mmol/L (≥200 mg/dl) or glycated hemoglobin (HbA_1c_) ≥48 mmol/mol (≥6.5%).^23^

### Ethics

The Northwest Multi-Centre Research Ethics Committee granted ethical approval for this study (REC reference 11/NW/0382) in 2011 and renewed it in 2016 (16/NW/0274) and 2021 (21/NW/0157). Study participants provided written informed consent.

### Study variables

#### The mediterranean lifestyle index (MEDLIFE)

Adherence to the MedDiet was evaluated at baseline (2009–2012), using the aforementioned Oxford WebQ. The estimated consumption of food groups and nutrients, as well as energy intake have been previously described.^24^ Other lifestyles, social habits, rest routine, sedentary behavior and physical activity were self-reported at baseline in the touchscreen questionnaire.

The MEDLIFE, representing adherence to a Mediterranean lifestyle, was evaluated according to the criteria established by Sotos-Prieto et al.,^25^ with minor adjustments conducted for the UK Biobank cohort.^18^ The MEDLIFE is comprised of three blocks. (1) “Mediterranean food consumption”, including 12 items about food intake (sweets, red meat, processed meat, eggs, legumes, white meat, fish/seafood, potatoes, low-fat dairy products, nuts, fruits, and vegetables); (2) “Mediterranean dietary habits”, consisting of 7 items on habits and practices concerning meals (wine, sugar sweetened beverages, coffee and tea consumption, use of salt, sodium to salt ratio, preference for whole-grains, and snacks); and (3) “Physical activity, rest, social habits, and conviviality”, with 6 items assessing physical activity (light, moderate or vigorous activity), collective sports, sedentary activities, social and family time, naps and sleep. However, given the unavailability of information in the UK Biobank, we could not include the consumption of olive oil and *sofrito* (a traditional sauce with olive oil, tomato, and onion), snacking outside meals, and eating with company. Nevertheless, previous studies have evaluated the impact of the modified MEDLIFE in the UK Biobank and have yielded consistent findings.^18^^,^^26^ Each item of the MEDLIFE scored 0 (non-adherent) or 1 point (adherent). Therefore, the final score ranged from 0 to 25 (highest adherence) (for a more detailed description of MEDLIFE, please see Supplementary Table S1).

#### Major adverse cardiovascular events (MACE), all-cause and CVD mortality

The primary outcomes of this study were the incidence of MACE, all-cause and CVD mortality. MACE was defined as a composite endpoint consisting of MI, IS, HF, revascularization procedures, and CVD death in accordance with the ESC Guidelines on CVD.^27^ Specifically, CVD was classified according to the 10th edition of the International Classification of Diseases (ICD-10) as follows: MI (I21–I23, I24.1, I25.2), IS (I63.0–I63.9), HF (I50, I50.0, I50.1 and I50.9), and CVD death (I0, I11, I13, I20–I79). The 4th version of the Office of Population Censuses and Surveys Classification of Interventions and Procedures (OPCS-4) was used to identify revascularization procedures, including coronary artery bypass graft surgery (CABG), specifically: K40 (K40.1–K40.9), K41 (K41.1–K41.9), K42 (K42.1–K42.9), K43 (K43.1–K43.9), K44 (K44.1–K44.9), K45 (K45.1–K45.9), K46 (K46.1–K46.9); and percutaneous coronary interventions (PCI): K49 (K49.1–K49.9), K50 (K50.1–K50.9) and K75 (K75.1–K75.9) (Supplementary Table S2). As in previous studies from the UK Biobank,^28^ CVD-related hospital admissions and death were identified by linkage to the Hospital Episode Statistics (HES) and the National Death Index, respectively. Hospital inpatient data was available up until October 2022 for England, until August 2022 for Scotland, and until May 2022 for Wales. Data on vital status and date of death based on central registers were obtained for England and Wales up to the 31st of May 2024, and up to the 31st of December 2023 for Scotland. Length of follow-up was estimated as time from the date of last dietary assessment (2009–2012) to the date of first MACE, death, or end of follow-up, whichever came first.

#### Other variables

Based on previous literature,^13^ and the directed acyclic graph (Supplementary Figure S1), we identified relevant confounders to be included in the models. Specifically, information on the following self-reported sociodemographic characteristics at baseline was used: age, sex, ethnicity (white or non-white), educational level (university or non-university), socio-economic status (townsend deprivation index) and family history of CVD. We also used data on health-related behaviors included smoking (never, former, or current), energy intake (kcal/day), vitamin or mineral supplement intake, as well as Body Mass Index (BMI), calculated as measured weight (kg) divided by the square of measured height (m^2^). Additionally, we considered the presence of comorbidities at baseline: hypertension (HTN) (defined as use of antihypertensive drugs, a self-reported diagnosis, a clinical record-based diagnosis as per ICD-10 codes, mean systolic blood pressure values ≥ 140 mmHg or diastolic blood pressure values ≥ 90 mmHg^29^), MACE (MI, IS, HF and revascularizations) and cancer (self-reported, general practice, and hospital admission records), as well as number of medications for CVD (beta-blockers, ACE inhibitors/ARA II, aspirin/antiplatelet, anticoagulants, calcium antagonists, diuretics, statins and other lipid lowering drugs). Lastly, we accounted for data regarding type 2 diabetes prognosis, including HbA_1c_ level (measured by high performance liquid chromatography analysis on a Bio-Rad VARIANT II Turbo), age at diagnosis, duration of diabetes (years), and use of type 2 diabetes medication (insulin and oral antidiabetics). Specifically, baseline HbA_1c_ concentrations were categorized as per ADA glycemic targets (<53 or ≥53 mmol/mol [7.0%]^30^) for individuals with diabetes. Duration of diabetes was calculated as the years between the first diagnosis of diabetes and the date of the last dietary assessment (end of baseline) for diagnosed patients, and assigned as 0 years for individuals identified through self-reporting, their HbA_1c_ or glucose levels.^13^

### Statistical analysis

**Fig. 1:**
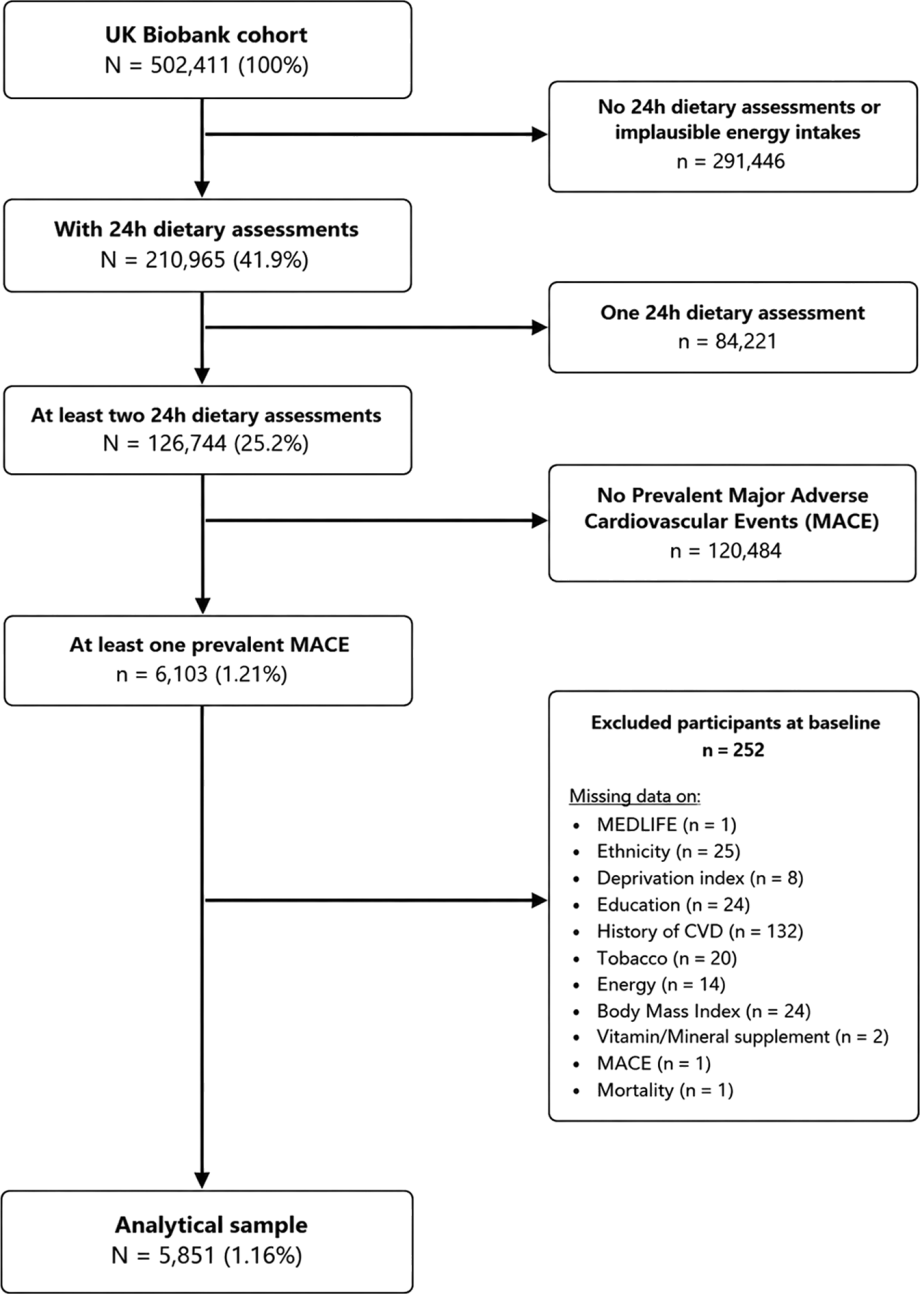
Flow diagram of included participants from the UK Biobank.

From the initial 5970 participants with prevalent type 2 diabetes at baseline who had a plausible dietary intake and 2 or more 24-h dietary assessments, we excluded those with type 1 diabetes or gestational diabetes at baseline (*n* = 736), missing more than two MEDLIFE items (*n* = 3), sociodemographic and lifestyle data (*n* = 228), BMI (*n* = 25) and type 2 diabetes related data (*n* = 1366). Thus, the final analytical sample comprised 3612 individuals (Fig. 1). Participants baseline main characteristics were summarized by sex-specific quartiles of adherence to the MEDLIFE using mean and standard deviation for continuous variables, and proportions for categorical ones.

Multivariable Cox proportional-hazard models using age as the underlying timescale were used to calculate hazard ratios (HRs), and their corresponding 95% confidence intervals (CI), of the association between the MEDLIFE and MACE, all-cause and CVD mortality. The MEDLIFE was categorized into sex-specific quartiles given that adherence to lifestyle behaviors typically differs between men and women,^31^ and to better distribute the study population (approximately one-quarter of participants in each group). The lowest quartile (representing the lowest adherence) was used as reference. We fitted four progressively adjusted models: Model 1, adjusted for sociodemographic variables (sex, ethnicity, educational level, deprivation index), family history of CVD, and region of assessment center; Model 2, further adjusted for lifestyle variables (smoking status, total energy intake, vitamin or mineral supplement use) and BMI; Model 3 additionally adjusted for potentially intermediate conditions (HTN, MACE, and cancer), and medications for CVD; and finally, Model 4 further adjusted for type 2 diabetes prognosis-related factors: HbA_1c_ control, age at diagnosis, diabetes duration, and type 2 diabetes medication. Model 4 was selected as the primary analysis model as it includes all prespecified variables that may influence the association of interest.

We also calculated *p-values* for linear trend by modeling the sex-specific quartiles of MEDLIFE as a continuous variable and evaluated the study associations per 2-point increment of the MEDLIFE, to provide a clinically interpretable estimate of change in adherence, consistent with previous studies.1^15^^,^^18^^,^^19^ Additionally, we assessed deviation from linearity by modeling the MEDLIFE as a restricted cubic spline with 3 knots set at the 10th, 50th and 90th percentiles (6, 8 and 12 points, respectively) and adjusted as in Model 4.

In secondary analyses, we assessed the independent association of the MEDLIFE blocks (per 2-point increment) with the main outcomes, adjusting as in Model 4 and additionally adjusting for the other blocks. Also, we evaluated the association of the MEDLIFE (per 2-point increment) with each MACE component (MI, IS, HF, and revascularization) to evaluate whether a particular event drove the association. Furthermore, to investigate potential effect modification, we conducted stratified analysis and tested interactions terms using likelihood ratio tests between the MEDLIFE and sex, ethnicity (non-white, white), education (university, non-university), deprivation index (under, equal or greater to the median), family history of CVD, smoking status (never, former, and current), vitamin or mineral supplement use (yes/no), BMI (under, equal or greater to the median), HTN (yes/no), MACE (yes/no), cancer (yes/no), medications for CVD (under, equal or greater to the median), HbA_1c_ control, age at diagnosis (under, equal or greater to the median), duration of diabetes (under, equal or greater to the median), and use of type 2 diabetes related medications (yes/no). Several variables were dichotomized at the median to facilitate interpretation and to ensure balanced group sizes.

Lastly, in sensitivity analyses, first, we replicated the main analysis including participants with at least three dietary assessments (n = 2161); second, we excluded the individuals with incident MACE during the initial two years of follow-up (n = 3449), to address potential reverse causality; third, we excluded participants with prevalent MACE at baseline (n = 3007); fourth, additionally adjusting for dyslipidemia; and fifth, we replicated main analysis for MACE and CVD mortality using the Fine–Gray regression model, where all-cause mortality excluding CVD mortality was the competing event.

The proportional hazards assumption was tested with the Schoenfeld residuals method (p > 0.05). Analyses were performed using Stata version 17 (Stata-Corp LLC, College Station, Texas). *P*-values were two-tailed and considered statistically significant at <0.05.

### Role of funding sources

The funding agencies had no role in study design, data collection and analysis, interpretation of results, manuscript preparation or in the decision to submit this manuscript for publication.

## Results

The baseline characteristics of the study participants are shown in Table 1. Study participants had a mean (SD) age of 59.8 (6.61) years, and 33.8% were women. The mean MEDLIFE score was 8.4 (range: 1–20) points in men, and 8.8 (range: 2–18) in women. Compared to participants in the lowest quartile of MEDLIFE adherence, those in the highest quartile were more likely to be older, female, non-smokers, and to have a higher socioeconomic and educational level, as well as a lower energy intake. Moreover, they had obesity and used type 2 diabetes medication less frequently, and more often had HbA_1c_ levels within the recommended range. Relative to the analytical sample, those excluded from the analyses due to missing information were more likely to be younger, female, and smokers. They also less frequently took type 2 diabetes medication and less often had family history of CVD (Supplementary Table S3).

**Table 1:** Baseline characteristics by MEDLIFE adherence (quartiles) among participants with type 2 diabetes.

	MEDLIFE				
	Total	Q1 (lowest)	Q2	Q3	Q4 (highest)
*n*	3612	1258 (34.8)	789 (21.8)	798 (22.1)	767 (21.2)
MEDLIFE^a^	8.56 (2.44)	6.01 (1.14)	8.25 (0.43)	9.57 (0.49)	12.0 (1.28)
Age, years	59.8 (6.61)	59.1 (6.77)	59.9 (6.64)	60.3 (6.49)	60.4 (6.34)
Sex, female (%)	1222 (33.8)	370 (29.4)	395 (50.1)	161 (20.2)	296 (38.6)
Ethnicity, n (%)					
White	3375 (93.4)	1198 (95.2)	737 (93.4)	742 (93.0)	698 (91.0)
Non-white	237 (6.6)	60 (4.8)	52 (6.6)	56 (7.0)	69 (9.0)
Education, n (%)					
University education	1330 (36.8)	411 (32.7)	261 (33.1)	307 (38.5)	351 (45.8)
Non-university education	2282 (63.2)	847 (67.3)	528 (66.9)	491 (61.5)	416 (54.2)
Deprivation index	−1.22 (3.04)	−1.02 (3.1)	−1.18 (3.07)	−1.49 (2.92)	−1.31 (3.05)
Family history of CVD, n (%)	2379 (65.9)	817 (64.9)	533 (67.6)	516 (64.7)	513 (66.9)
Region of assessment, n (%)					
England	3334 (92.3)	1148 (91.3)	715 (90.6)	734 (91.9)	737 (96.1)
Wales	130 (3.60)	55 (4.4)	29 (3.7)	34 (4.26)	12 (1.56)
Scotland	148 (4.10)	55 (4.3)	45 (5.7)	30 (3.76)	18 (2.35)
Smoking status, n (%)					
Never	1583 (43.8)	517 (41.1)	356 (45.1)	335 (41.9)	375 (48.9)
Former	1756 (48.6)	614 (48.8)	366 (46.4)	419 (52.5)	357 (46.5)
Current	273 (7.56)	127 (10.1)	67 (8.49)	44 (5.5)	35 (4.6)
Energy intake (Kcal/day)	2043.2 (521.3)	2135.7 (535.7)	1978.4 (499.9)	2057.8 (515.5)	1943.0 (498.1)
Vitamin/Mineral supplement, n (%)	1691 (46.8)	530 (42.1)	393 (49.8)	362 (45.4)	406 (52.9)
Body Mass Index (kg/m^2^)^e^, n (%)					
<30.3	1806 (50.0)	514 (40.9)	374 (47.4)	452 (56.6)	466 (60.8)
≥30.3	1806 (50.0)	744 (59.1)	415 (52.6)	346 (43.4)	301 (39.2)
Hypertension, n (%)	3051 (84.5)	1077 (85.6)	659 (83.5)	694 (87.0)	621 (81.0)
Major adverse cardiovascular events^b^, n (%)	605 (16.8)	224 (17.8)	131 (16.6)	145 (18.2)	105 (13.7)
Cancer, n (%)	462 (12.8)	164 (13.0)	96 (12.2)	99 (12.4)	103 (13.4)
Number of cardiovascular disease medications^c^	2.42 (1.47)	2.53 (1.47)	2.41 (1.44)	2.46 (1.50)	2.19 (1.45)
HbA_1c_ (mmol/mol), n (%)					
<53	2372 (65.7)	771 (61.3)	509 (64.5)	549 (68.8)	543 (70.8)
≥53	1240 (34.3)	487 (38.7)	280 (35.5)	249 (31.2)	224 (29.2)
Age at diagnosis, n (%)^e^					
<55	1734 (48.1)	668 (53.1)	371 (47.0)	362 (45.4)	333 (43.4)
≥55	1878 (52.0)	590 (46.9)	418 (53.0)	436 (54.6)	434 (56.6)
Duration (years) of Type 2 Diabetes, n (%)^e^					
<5.92	1806 (50.0)	594 (47.2)	414 (52.5)	395 (49.5)	403 (52.5)
≥5.92	1806 (50.0)	664 (52.8)	375 (47.5)	403 (50.5)	364 (47.5)
Type 2 Diabetes Medications^d^, n (%)	2436 (67.4)	898 (71.4)	543 (68.8)	505 (63.3)	490 (63.9)

Continuous variables are expressed as mean (standard deviation). Categorical variables are expressed as percentages (%).

MEDLIFE, Mediterranean Lifestyle Index; Q, quartiles.

a MEDLIFE range: Q1: 1–7; Q2: 8–9; Q3: 9–10, Q4: 11–20.

b Myocardial infarction, ischemic stroke, heart failure and revascularization.

c Beta-blocker, ACE inhibitors/ARA-II, aspirin/antiplatelet, anticoagulants, calcium antagonists, diuretics, statins and lipid lowering drugs.

d Insulin and oral antidiabetics.

e Categorized by the median.

**Fig. 2:**
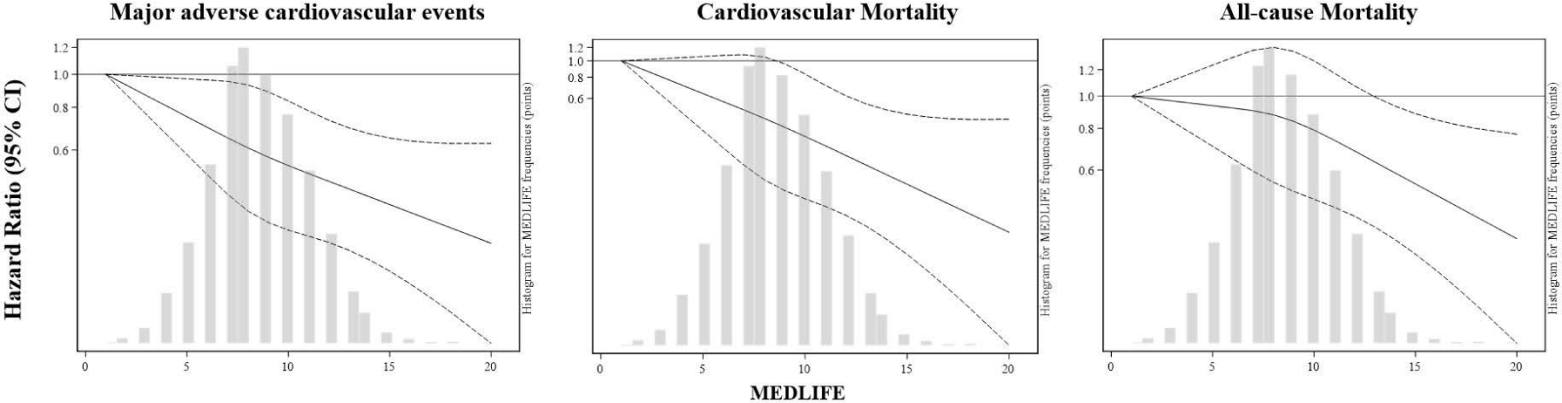
Restricted cubic spline regression for the association of the MEDLIFE with risk of major adverse cardiovascular events, cardiovascular, and all-cause mortality Adjusted for sex, ethnicity (white, non-white), education (university, non-university education), deprivation index, family history of cardiovascular disease, region of assessment, smoking status (never, past, and current smoker), energy intake (Kcal), vitamin/mineral supplement (yes/no), Body Mass Index (under, equal or over the median), hypertension (yes/no), prevalent MACE (yes/no), cancer (yes/no), number of medications for CVD, HbA1c (<53 or ≥53 mmol/mol), age at T2D diagnosis (under, equal or over the median), duration of diabetes (under, equal or over the median), and medications for T2D (yes/no) Abbreviations: MEDLIFE, Mediterranean Lifestyle Index; CI, Confidence Interval. The 3 knots were set at the 10th, 50th and 90th percentiles (6, 8 and 12 points, respectively). The histogram displays the frequency of scores within the MEDLIFE range (0–25). p for non-linearity >0.05.

After a median follow-up of 10.5–12.1 years, 737 (20.4%) incident MACE, 645 (17.9%) all-cause and 171 (4.73%) CVD deaths were identified. Higher adherence to the MEDLIFE was associated with a progressively lower risk of MACE, all-cause and CVD mortality. Specifically, compared to the lowest quartile of MEDLIFE, the HR (95% CI) for MACE in model 4 was 0.95 (0.78–1.16) for the second, 0.75 (0.61–0.91) for the third, and 0.73 (0.59–0.91) for the highest quartile (p-trend <0.001). Similarly, when comparing the first vs the fourth quartile of MEDLIFE, the HR (95% CI) for all-cause mortality was 0.74 (0.58–0.93); p-trend <0.01. This association was even more pronounced for CVD mortality, 0.49 (0.30–0.81) (p-trend <0.001). Accordingly, every 2-point increment of MEDLIFE adherence was associated with a 12% lower risk of MACE, an 9% lower risk of all-cause death, and a 21% lower risk of CVD death (all p-values <0.01) (Table 2). Spline analyses further corroborate the linearity of these associations (p for non-linearity >0.05) (Fig. 2).

**Table 2:** Hazard ratios (95% confidence interval) for major adverse cardiovascular events, cardiovascular, and all-cause mortality by MEDLIFE adherence (quartiles).

	MEDLIFE					
	Q1 (lowest)	Q2	Q3	Q4 (highest)	p-trend	Per 2-point increment
MEDLIFE	6.01 (1.14)	8.25 (0.43)	9.57 (0.49)	12.0 (1.28)		
Major adverse cardiovascular events						
Cases/N	292/1258	167/789	151/798	127/767		737/3612
Person-years	11,409.5	7360.1	7456.4	7365.7		33,591.6
Model 1^a^	1 Ref.	0.97 (0.80–1.17)	0.71 (0.58–0.86)	0.67 (0.54–0.82)	p < 0.001	0.85 (0.80–0.91)
Model 2^b^	1 Ref.	0.96 (0.79–1.17)	0.73 (0.60–0.89)	0.68 (0.55–0.84)	p < 0.001	0.86 (0.81–0.91)
Model 3^c^	1 Ref.	0.94 (0.77–1.14)	0.73 (0.59–0.89)	0.72 (0.58–0.89)	p < 0.001	0.87 (0.82–0.93)
Model 4^d^	1 Ref.	0.95 (0.78–1.16)	0.75 (0.61–0.91)	0.73 (0.59–0.91)	p < 0.001	0.88 (0.83–0.94)
All-cause mortality						
Cases/N	251/1258	140/789	146/798	108/767		645/3612
Person-years	14,102.5	9000.0	9033.9	8895.0		41,031.5
Model 1^a^	1 Ref.	0.91 (0.74–1.12)	0.80 (0.65–0.98)	0.65 (0.52–0.82)	p < 0.001	0.87 (0.82–0.93)
Model 2^b^	1 Ref.	0.93 (0.75–1.14)	0.85 (0.69–1.04)	0.69 (0.55–0.88)	p < 0.01	0.90 (0.84–0.96)
Model 3^c^	1 Ref.	0.93 (0.75–1.14)	0.85 (0.69–1.04)	0.72 (0.57–0.91)	p < 0.01	0.91 (0.85–0.97)
Model 4^d^	1 Ref.	0.93 (0.76–1.15)	0.85 (0.69–1.05)	0.74 (0.58–0.93)	p < 0.01	0.91 (0.85–0.98)
Cardiovascular mortality						
Cases/N	70/1258	48/789	32/798	21/767		171/3612
Person-years	14,102.5	9000.0	9033.9	8895.0		41,031.5
Model 1^a^	1 Ref.	1.21 (0.83–1.75)	0.60 (0.40–0.92)	0.44 (0.27–0.73)	p < 0.001	0.76 (0.67–0.87)
Model 2^b^	1 Ref.	1.21 (0.83–1.76)	0.63 (0.41–0.96)	0.46 (0.28–0.75)	p < 0.001	0.77 (0.68–0.88)
Model 3^c^	1 Ref.	1.18 (0.81–1.71)	0.62 (0.41–0.95)	0.48 (0.29–0.80)	p < 0.001	0.78 (0.69–0.89)
Model 4^d^	1 Ref.	1.20 (0.82–1.74)	0.62 (0.40–0.96)	0.49 (0.30–0.81)	p < 0.001	0.79 (0.69–0.90)

Values of all models are hazard ratios (95% confidence interval).

MEDLIFE, Mediterranean Lifestyle Index; Q, quartiles.

a Adjusted for sex, ethnicity (white, non-white), education (university, non-university education), deprivation index, family history of cardiovascular disease (yes/no), and region of assessment.

b Adjusted as for Model 1 + smoking status (never, past, and current smoker), energy intake (Kcal), vitamin/mineral supplement (yes/no) and Body Mass Index (under/equal or over the median).

c Adjusted as for Model 2 + hypertension (yes/no), prevalent MACE (yes/no), cancer (yes/no), and number of medications for CVD.

d Adjusted as for Model 3 + HbA_1c_ (<53 or ≥53 mmol/mol), age at T2D diagnoses (under, equal or over the median), duration of diabetes (under, equal or over the median), and medications for T2D (yes/no).

**Fig. 3:**
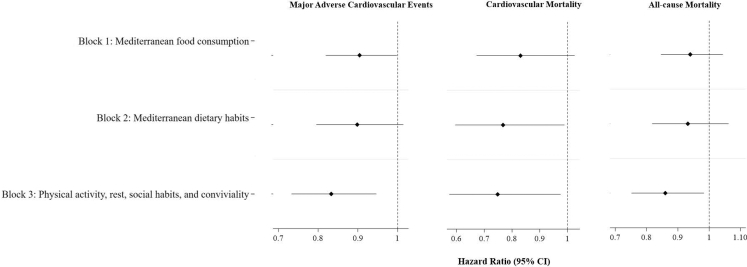
Association of each MEDLIFE block with risk of major adverse cardiovascular events, cardiovascular, and all-cause mortality Adjusted for sex, ethnicity (white, non-white), education (university, non-university education), deprivation index, family history of cardiovascular disease, region of assessment, smoking status (never, past, and current smoker), energy intake (Kcal), vitamin/mineral supplement (yes/no), Body Mass Index (under, equal or over the median), hypertension (yes/no), prevalent MACE (yes/no), cancer (yes/no), number of medications for CVD, HbA1c (<53 or ≥53 mmol/mol), age at T2D diagnosis (under, equal or over the median), duration of diabetes (under, equal or over the median), medications for T2D (yes/no), and the MEDLIFE minus each block being evaluated. Abbreviations: MEDLIFE Mediterranean Lifestyle Index, CI Confidence Interval.

In secondary analyses, we found that each MEDLIFE block contributed significantly to the observed associations. Every 2-point increment in the score of “Mediterranean food consumption block” was associated with a 10% lower risk of MACE (HR: 0.90; 95% CI: 0.82–0.99; p-value = 0.046). The second MEDLIFE block, representing mediterranean dietary habits, showed a 23% lower risk of CVD mortality (HR: 0.77, 95% CI: 0.60–0.99; p-value = 0.041). Notably, adherence to the third block, comprising physical activity, rest, social habits, and conviviality, was associated with a lower risk of all three outcomes. Specifically, the HR (95% CI) was 0.83 (0.73–0.95) for MACE (p-value <0.01), 0.86 (0.75–0.98) for all-cause mortality (p-value = 0.027), and 0.75 (0.57–0.97) for CVD mortality (p-value = 0.032) (Fig. 3). In addition, when we individually evaluated each MACE, we found that although the direction of the association was consistent across all outcomes (MI, IS, HF and revascularization), adherence to the MEDLIFE was associated with lower risk of HF (HR per 2-point increase: 0.90, 95% CI: 0.81–1.00 (p-value: 0.056)) (Supplementary Figure S2). Regarding population subgroups, no significant interactions were found but for educational level and medications for type 2 diabetes. Notably, higher adherence to the MEDLIFE was significantly associated with a lower risk of MACE in individuals taking medication for type 2 diabetes or with a lower educational level (Supplementary Table S4).

Lastly, sensitivity analysis yielded similar results when: 1) including participants with three or more dietary questionnaires (Supplementary Table S5), 2) excluding the first two years of follow-up (Supplementary Table S6), 3) when participants with MACE at baseline were excluded, although the association did not reach statistical significance for CVD mortality (Supplementary Figure S3), 4) when we additionally adjusted for prevalent dyslipidemia at baseline (Supplementary Table S7); and 5) when using the Fine–Gray regression model (Supplementary Table S8).

## Discussion

In this prospective cohort of British adults with type 2 diabetes, higher adherence to the MEDLIFE was associated with stepwise decreases in MACE risk, all-cause and CVD mortality. Compared to participants in the lowest quartile of MEDLIFE, those in the highest quartile had a 27% lower risk of MACE, a 26% lower risk of all-cause death, and a 51% lower risk of CVD death. These associations were independent of multiple diabetes-related prognostic factors. Each MEDLIFE block significantly contributed to the observed associations, underscoring the importance of a comprehensive healthy lifestyle. The findings remained robust in sensitivity analyses.

Optimal management of individuals with type 2 diabetes, including comprehensive lifestyle and drug therapy has shown to improve the trajectory of the disease.^12^ Earlier observational studies among individuals with type 2 diabetes with characteristics similar to ours (predominantly middled aged men, overweight or obese with low use of type 2 diabetes medications) have shown that a healthy lifestyle, defined as per several scoring criteria, is associated with a lower risk of CVD and all-cause mortality,^12^^,^^13^ consistent with the findings of the current study. A previous analysis from the UK Biobank showed that for each 1-point increase in adherence to a healthy lifestyle (no current smoking, moderate alcohol consumption, regular physical activity, healthy diet, less sedentary behavior, and adequate sleep and social connection), the risk for CVD mortality was decreased by 11%.^13^ Similarly, a pooled analysis of five cohorts found that for each additional lifestyle factor (no current smoking, low-moderate alcohol consumption, regular physical activity, healthy diet, and optimal body weight) individuals with type 2 diabetes had a 12% lower risk of incident CVD.^12^ However, the risk for MACE remains high even among individuals with well-managed type 2 diabetes, highlighting the need for additional scalable tools for clinical practice to address this burden.^4^ Adherence to the MEDLIFE, a comprehensive lifestyle index integrating multiple behavioral domains has been associated with a 30% lower risk of type 2 diabetes in a previous study involving general participants in the UK Biobank.^18^ Moreover, adherence to the MEDLIFE has been associated with a lower risk of MACE^16^ and reversion of metabolic syndrome^17^ in patients with established CVD. However, no study had yet evaluated the association of the MEDLIFE with MACE in individuals with type 2 diabetes. Our findings build upon the aforementioned evidence, indicating that adherence to the MEDLIFE may lower the risk of the predominant adverse outcomes in type 2 diabetes, comparable to that observed in the general population, even beyond the Mediterranean region, supporting its potential as a scalable tool for clinical practice.

The COURAGE (Clinical Outcomes Utilizing Revascularization and Aggressive Drug Evaluation) trial,^32^ a reflection of the difficulty in controlling risk factors in patients with diabetes and CVD, described achievement of an HbA_1c_ of <7%, and lifestyle factors—smoking cessation, physical activity, and adherence to dietary advice—as the most prominent cornerstones of diabetes management. In accordance with these objectives, and as previously reported in observational^33^ and experimental^6^ studies, we observed that adherence to the MedDiet block was individually associated with a lower risk of MACE. Indeed, the MedDiet is considered an exemplary dietary pattern for patients with type 2 diabetes.^7^ Underlying mechanisms include improvements in metabolic function, such as glycemic and lipid control, achieved through the consumption of foods with high polyphenol and monounsaturated fat content, as well as reduced inflammation and oxidative stress, the hallmark of type 2 diabetes. Furthermore, accompanying the MedDiet with additional healthy dietary practices (no sugar-sweetened beverage consumption, preference for whole-grain cereal, or no salt at meals) may potentiate the benefits of the MedDiet.^34^ Apart from dietary considerations, physical activity enhances insulin sensitivity through weight control and muscle strengthening in individuals with type 2 diabetes,^8^ whereas insufficient or short sleep duration has been associated with adverse effects on serum lipids and insulin resistance.^4^ Thus, adherence to the third MEDLIFE block, emphasizing not only physical activity and optimal rest, but also strong social connections, may lower the risk of MACE, overall and CVD mortality.

Notably, we found that the observed associations were independent of prognostic factors such as HbA_1c_ control, duration of the disease, age at diagnosis, and pharmacological treatment, highlighting the potential of the MEDLIFE across the clinical spectrum of diabetes. Several trials—the UKPDS^35^ (UK Prospective Diabetes Study), ACCORD^36^ (Action to Control Cardiovascular Risk in Diabetes), ADVANCE^37^ (Action in Diabetes and Vascular disease: preterAx and diamicroN-MR Controlled Evaluation) and VADT^38^(Veterans Affairs Diabetes Trial)—have evaluated diverse pharmacological and behavioral interventions on MACE and mortality, and highlighted the importance of early glucose and blood pressure control in type 2 diabetes patients. Interestingly, we observed that adherence to the MEDLIFE was associated with a substantially lower risk of MACE in individuals who took type 2 diabetes medication, which reinforces the role of a healthy lifestyle even when using drug treatment.^39^ Taken together, medication for T2D and adherence to the MEDLIFE may substantially lower the risk for MACE, highlighting their additive benefits.

The association with the MEDLIFE showed a consistent trend across all MACE endpoints (MI, IS, HF, and revascularization), nearly reaching statistical significance for HF. The risk for HF in type 2 diabetes patients persists somewhat elevated even after optimal control of traditional risk factors such as HTN, smoking, hyperlipidemia and hyperglycemia.^40^ In individuals with type 2 diabetes, HF is strongly driven by the underlying cardiac remodeling, diastolic dysfunction and metabolic derangements, such as insulin resistance, often attributed to obesity,^40^ which can be controlled via healthy lifestyle practices, as captured by the MEDLIFE. Importantly, over 85% of the study population was overweight or obese. In the Look AHEAD trial,^41^ a post hoc analysis indicated that a sustained, long-term improvement in weight loss and cardiorespiratory fitness was associated with lower risk of HF. Moreover, regular physical activity has been linked to lower levels of high-sensitivity cardiac troponin T (hs-cTnT),^42^ a potent predictor of HF.^43^ In addition, adherence to a MedDiet has been associated with a lower risk of HF clinical biomarkers including N-terminal pro-B-type natriuretic peptide, oxidized low-density lipoprotein cholesterol, and lipoprotein (a) in the PREDIMED trial.^6^

Several strengths characterized this study, including the prospective design, long follow-up, the use of repeated 24-h dietary assessments, and the reliability of health and outcome data. Nevertheless, several limitations should be noted. First, diet and other health behaviors were self-reported, thus, exposure assessment may be subject to misclassification and recall bias. Second, several components of the MEDLIFE could not be accounted for; nevertheless, previous studies from the UK Biobank1^18^^,^^26^ have reported findings consistent with those from other studies1^14^^,^^15^ in which these components were included. This suggests that overall lifestyle patterns, rather than individual practices alone, may have the greatest impact on disease prevention. The absence of these components may have led to a conservative estimation of the associations. Third, changes in lifestyle could not be assessed due to the progressively lower number of participants over follow-up. Thus, positive shifts in health behaviors after type 2 diabetes diagnosis could not be assessed. Fourth, despite extensive adjustment for variables influencing lifestyle, health, and type 2 diabetes progression, some residual confounding cannot be ruled out given the observational nature of the study and causal inferences remain limited. In addition, some degree of overadjustment may have occurred, as adjustment for variables on the causal pathway could have attenuated the observed associations, potentially leading to conservative estimates. Fifth, the UK Biobank recruited volunteers, which may limit the generalizability of the findings. In addition, the exclusion of participants with missing data may have introduced selection bias, as included individuals differed from those excluded, potentially influencing the observed associations. Sixth, two dietary assessments may not reflect habitual dietary intake, yet our results remain robust among participants having three or more dietary assessments. Lastly, this study comprised a British population, which may limit the generalizability to other populations.

In conclusion, higher adherence to the MEDLIFE—a MedDiet accompanied by healthy dietary habits, optimal rest, physical activity and social engagement—was associated with a substantially lower risk of MACE, all-cause and CVD mortality in British individuals with type 2 diabetes. Strategies to adopt healthy behaviors in individuals with diabetes are crucial; our findings highlight the MEDLIFE as a promising tool for type 2 diabetes management and further studies should further address this in routine clinical care settings.

## Contributors

MDCAR, MSP, and FRA formulated the study question and design. MDCAR researched data, conducted the formal analyses, and wrote the manuscript. MSP contributed to discussion, analyzed data, edited the manuscript, and supervised the project. FRA contributed to the discussion, reviewed and edited the manuscript and supervised the project. MDCAR, JDG, SNK, MRC, ACC, BF, FRA, and MSP contributed to results interpretation, edited the manuscript and approved the final version. MDCAR and MSP accessed and verified all data in the manuscript. All authors had full access to the data in the study and accept responsibility for the decision to submit for publication. All authors had full access to the data in the study and accept responsibility for the decision to submit for publication.

## Data sharing statement

The data used in this study are available from the UK Biobank, subject to their data access policies. Interested researchers can apply for access at https://www.ukbiobank.ac.uk and are required to cover the access fees.

## Declaration of interests

Authors declare no conflict of interest.
