# Supplementary material for: Mediterranean lifestyle and risk of adverse cardiovascular events, all-cause and cardiovascular mortality among individuals with type 2 diabetes
Source: eClinicalMedicine. 2026 Aug 7;98:104134. doi: 10.1016/j.eclinm.2026.104134 (PMC13475649; doi:10.1016/j.eclinm.2026.104134)
Supplement: Supplementary file 1 [file mmc1.docx]

**SUPPLEMENTARY TABLES AND FIGURES**

**Title: Mediterranean lifestyle and risk of Adverse Cardiovascular Events, all-cause and Cardiovascular Mortality among Individuals with Type 2 Diabetes**

**Authors**: María del Carmen Aznar de la Riera^1,2^, MSc; Jesús Díaz-Gutiérrez^3,4^, MD; Stefanos N. Kales^5,6^, MD, PhD; Miguel Ruiz-Canela^7,8,9^, PhD; Almudena Castro Conde^10^, MD, PhD; Beth Frates^11^, MD, PhD; Fernando Rodríguez-Artalejo^1,2,12^ MD, PhD; Mercedes Sotos-Prieto^1,2,5,12^, PhD

^1^ Department of Preventive Medicine and Public Health. School of Medicine. Universidad Autónoma de Madrid, Calle del Arzobispo Morcillo, 4. 28029, Madrid, Spain.

^2^ CIBERESP (CIBER of Epidemiology and Public Health), Av. Monforte de Lemos, 3-5. 28029, Madrid, Spain.

^3^ Sant Pau Research Institute (IR SANT PAU), Barcelona, Spain

^4^ Department of Cardiology, Hospital de la Santa Creu i Sant Pau, Barcelona, Spain

^5^ Department of Environmental Health, Harvard T.H. Chan School of Public Health, 665 Huntington Avenue. Boston, Massachusetts 02115, USA.

^6^ Occupational Medicine, The Cambridge Health Alliance/ Harvard Medical School

^7^ Biomedical Research Center Network on Physiopathology of Obesity and Nutrition (CIBEROBN), Institute of Health Carlos III, Madrid, Spain

^8^ University of Navarra, Department of Preventive Medicine and Public Health, Institute of Nutrition and Health, Pamplona, Navarra, Spain.

^9^ IdiSNA, Navarra Institute for Health Research, Pamplona, Spain.

^10^ Department of Cardiology, University Hospital La Paz, IdiPAZ, Biomedical Research Center-Cardiovascular Diseases (CIBERCV-ISCIII), 28046 Madrid, Spain.

^11^ Harvard Medical School, Associate Professor, Part-Time, Department of Physical Medicine and Rehabilitation, Spaulding Rehabilitation Hospital Charlestown, MA, USA.

^12^ IMDEA-NUTRITION Institute. CEI UAM+CSIC, Ctra. de Canto Blanco 8, E. 28049, Madrid, Spain.

**Corresponding author:** Mercedes Sotos-Prieto, PhD

Department of Preventive Medicine and Public Health, School of Medicine, Universidad Autónoma de Madrid, Calle del Arzobispo Morcillo 4, 28029 Madrid, Spain. Telephone: (+34) 91 497 54 41. E-mail: [mercedes.sotos@uam.es](mailto:mercedes.sotos@uam.es)

**SUPPLEMENTARY TABLES AND FIGURES:**

**Supplementary Table S1.** Description of the Mediterranean Lifestyle (MEDLIFE) index, adapted to the UK Biobank

**Supplementary Table S2**. Description of Major Adverse Cardiovascular Events

**Supplementary Table S3**. Baseline characteristics of the UK Biobank participants by inclusion status in the analytical sample.

**Supplementary Table S4.** Hazard ratios (95% confidence interval) for major adverse cardiovascular events by MEDLIFE adherence (quartiles) according to subgroups of sociodemographic and clinical characteristics

**Supplementary Table S5.** Hazard Ratios (95% confidence interval) for major adverse cardiovascular events, cardiovascular and all-cause mortality by MEDLIFE adherence (quartiles) in participants with ≥three dietary assessments

**Supplementary Table S6.** Hazard Ratios (95% confidence interval) for major adverse cardiovascular events, cardiovascular and all-cause mortality by MEDLIFE adherence excluding participants who suffered a MACE during the initial two years of follow-up

**Supplementary Table S7.** Hazard ratios (95% confidence interval) for major adverse cardiovascular events, cardiovascular, and all-cause mortality by MEDLIFE adherence (quartiles) additionally adjusting for dyslipidemia

**Supplementary Table S8.** Hazard ratios (95% confidence interval) for major adverse cardiovascular events, cardiovascular, and all-cause mortality by MEDLIFE adherence (quartiles) using the Fine-Gray method.

**Figure S1.** Directed Acyclic Graph of the total effects of the MEDLIFE on MACE

**Figure S2.** Association of the MEDLIFE with the individual major adverse cardiovascular events

**Figure S3.** Restricted cubic spline regression for association of the MEDLIFE with risk of major adverse cardiovascular events (MACE), cardiovascular, and all-cause mortality excluding participants with a previous MACE (N=3,003)

**Supplementary Table S1.** Description of the Mediterranean Lifestyle (MEDLIFE) index, adapted to the UK Biobank

| **Index items** | **Components (serving size)** | **Criterion for 1 point** | **% scoring 1 point** |
| --- | --- | --- | --- |
| Block 1: *Mediterranean food consumption* | | |  |
| 1. Sweets | Cookies, chocolate cookies, pastries, donuts, homemade baked goods, store-bought baked goods, muffins (50 g), chocolates (30 g). | ≤ 2 serv/wk | 22.6 |
| 2. Red meat | Beef, pork, lamb (125 g). | < 2 serv/wk | 45.6 |
| 3. Processed meat | Sausage, soft spicy sausage, bacon, cured ham, cooked ham, hamburger, liver, organ meats, pâté (42.5 g). | ≤ 1 serv/wk | 36.3 |
| 4. Eggs | Eggs (1 unit = 65 g). | ≥ 2 to ≤ 4 serv/wk | 14.5 |
| 5. Legumes | Lentils, beans, chickpeas, peas (150 g cooked). | ≥ 3 serv/wk | 11.7 |
| 6. White meat | Chicken/turkey with skin, chicken/turkey without skin, rabbit (125 g). | ≤ 2 serv/wk | 62.5 |
| 7. Fish/seafood | White fish and tinned tunned, fatty fish, codfish, salted or smoked fish, sushi, and shellfish (125 g). | ≥ 3 serv/wk | 22.3 |
| 8. Potatoes | Baked or boiled potatoes (150 g). | ≤ 2 serv/wk | 20.5 |
| 9. Low-fat dairy products | Skim milk, low-fat milk (200 ml), low fat yogurt (125 g), fresh soft cheese (50 g). | 1.5-2.5 serv/d | 27.7 |
| 10. Nuts | Almonds, peanuts, hazelnuts, walnuts, nut based spreads (30 g). | ≥ 3 serv/wk | 19.6 |
| 11. Sofrito | Sauce of olive oil with onion, pepper, other vegetables (250 g) and tomato (150 g). | > 2/4 ingredients above the median | -- |
| 12. Fruit | Orange, banana, apple, pear, kiwi, mango, avocado, peach, apricot, nectarine, clementine, strawberry, cherries, plums, figs, grapes, watermelon, melon (160 g), dates and dried fruits (30 g). | ≥ 3 serv/d | 9.72 |
| 13. Vegetables | Spinach, cauliflower, broccoli, lettuce, carrot, squash, green beans, eggplant, zucchini, cucumber, pepper, asparagus, gazpacho, garden salad, tomato, other vegetables (236.59 g for green leafy, half for others) *(excluding potatoes).* | ≥ 2 serv/d | 21.0 |
| 14. Olive oil | Olive oil (1 Tbsp = 13.5 g) | ≥ 3 serv/d | --- |
| Block 2: *Mediterranean* d*ietary habits* | | | |
| 15. Wine | Red/white wine (1 glass, 100 ml). | Women: > 0 to ≤ 1 serv/d  Men: > 0 to ≤ 2 serv/d | 23.9 |
| 16. Limit salt at meals | Do you add salt to your food? (*After cooking*). | “Never” or “Sometimes” | 86.7 |
| 17. Low salt consumption | Low sodium-to-potassium ratio and low sodium consumption. | Na/K ratio ≤ 0.57  Na ≤ 2.000 mg/d | 39.4 |
| 18. Preference for whole grain products | Fiber from whole grain cereals. | > 6 g/d fiber from cereals | 14.0 |
| 19. Snacks | Potato chips, popcorn, or other chips (50 g). | ≤ 1 serv/wk | 57.6 |
| 20. Limit snacking between meals | Do you tend to snack in between meals or before going to bed? | No | --- |
| 21. Healthy beverages consumption | Coffee, decaffeinated coffee (1 cup = 50 ml) or tea (1 cup = 250 ml). | ≥ 1 to ≤ 4 serv/d | 64.7 |
| 22. Limited consumption of sugar-sweetened beverages | Sugar-sweetened beverages + juice (150 ml). | ≤ 1/wk | 21.9 |
| Block 3: *Physical activity, rest social habits and conviviality* | | | |
| 23. Physical activity | Any light, moderate, or vigorous physical activity | Any light, moderate, or vigorous physical activity | 90.6 |
| 24. Nap | Napping throughout the week. | “Usually” or “Sometimes” if night-time sleep is adequate | 20.3 |
| 25. Hours of sleep | Sleeping throughout the week. | 6-8 h/d | 39.5 |
| 26. Limit sedentary activities | Watching TV, using internet or driving. | ≤ 2 h/d | 7.72 |
| 27. Eating in company | Do you have lunch or dinner with friends, family, or others? | Yes | --- |
| 28. Collective sports | Playing soccer, tennis, squash, basketball, or other team sports, running in group, jogging; etc. | ≥ 1 h/wk | 40.3 |
| 29. Socializing with friends or family | Frequency on what you see family (different from whom you live with) or friends (home, church, bars…). | ≥ 3 times/wk | 39.6 |

Abbreviations: *N*, total number of participants; serv, serving; d, day, wk, week; Tbsp, tablespoons; TV, television.

*The items in gray are those that could not be accounted in the UK Biobank.

**Supplementary Table S2**. Description of Major Adverse Cardiovascular Events

| **MACE** | |
| --- | --- |
| Myocardial Infarction (ICD-10) | I21 Acute myocardial infarction   - I21.0 Acute transmural myocardial infarction of anterior wall - I21.1 Acute transmural myocardial infarction of inferior wall - I21.2 Acute transmural myocardial infarction of other sites - I21.3 Acute transmural myocardial infarction of unspecified site - I21.4 Acute subendocardial myocardial infarction - I21.9 Acute myocardial infarction, unspecified |
|  | I22 Subsequent myocardial infarction   - I22.0 Subsequent myocardial infarction of anterior wall - I22.1 Subsequent myocardial infarction of inferior wall - I22.8 Subsequent myocardial infarction of other sites - I22.9 Subsequent myocardial infarction of unspecified site |
|  | I23 Complications following acute myocardial infarction   - I23.0 Haemopericardium as current complication following acute myocardial infarction - I23.1 Atrial septal defect as current complication following acute myocardial infarction - I23.2 Ventricular septal defect as current complication following acute myocardial infarction - I23.3 Rupture of cardiac wall without haemopericardium as current complication following acute myocardial infarction - I23.5 Rupture of papillary muscle as current complication following acute myocardial infarction - I23.6 Thrombosis of atrium, auricular appendage and ventricle as current complications following acute myocardial infarction - I23.8 Other current complications following acute myocardial infarction |
|  | I24.1 Dressler's syndrome |
|  | I25.2 Old myocardial infarction |
| Heart Failure  (ICD-10) | I50 Heart failure   - I50.0 Congestive heart failure - I50.1 Left ventricular failure - I50.9 Heart failure, unspecified |
| Ischemic Stroke  (ICD-10) | I63 Cerebral infarction   - I63.0 Cerebral infarction due to thrombosis of precerebral arteries - I63.1 Cerebral infarction due to embolism of precerebral arteries - I63.2 Cerebral infarction due to unspecified occlusion or stenosis of precerebral arteries - I63.3 Cerebral infarction due to thrombosis of cerebral arteries - I63.4 Cerebral infarction due to embolism of cerebral arteries - I63.5 Cerebral infarction due to unspecified occlusion or stenosis of cerebral arteries - I63.6 Cerebral infarction due to cerebral venous thrombosis, nonpyogenic |
|  | - I63.8 Other cerebral infarction - I63.9 Cerebral infarction, unspecified |
| Coronary Revascularization  (OPCS-4) | **Percutaneous coronary intervention (PCI)**  K49 Transluminal balloon angioplasty of coronary artery   - K49.1 Percutaneous transluminal balloon angioplasty of one coronary artery - K49.2 Percutaneous transluminal balloon angioplasty of multiple coronary arteries - K49.3 Percutaneous transluminal balloon angioplasty of bypass graft of coronary artery - K49.4 Percutaneous transluminal cutting balloon angioplasty of coronary artery - K49.8 Other specified transluminal balloon angioplasty of coronary artery - K49.9 Unspecified transluminal balloon angioplasty of coronary artery   K50 Other therapeutic transluminal operations on coronary artery   - K50.1 Percutaneous transluminal laser coronary angioplasty - K50.2 Percutaneous transluminal coronary thrombolysis using streptokinase - K50.3 Percutaneous transluminal injection of therapeutic substance into coronary artery NEC - K50.4 Percutaneous transluminal atherectomy of coronary artery - K50.8 Other specified other therapeutic transluminal operations on coronary artery - K50.9 Unspecified other therapeutic transluminal operations on coronary artery   K75 Percutaneous transluminal balloon angioplasty and insertion of stent into coronary artery   - K75.1 Percutaneous transluminal balloon angioplasty and insertion of 1-2 drug-eluting stents into coronary artery - K75.2 Percutaneous transluminal balloon angioplasty and insertion of 3 or more drug-eluting stents into coronary artery - K75.3 Percutaneous transluminal balloon angioplasty and insertion of 1-2 stents into coronary artery - K75.4 Percutaneous transluminal balloon angioplasty and insertion of 3 or more stents into coronary artery NEC - K75.8 Other specified percutaneous transluminal balloon angioplasty and insertion of stent into coronary artery - K75.9 Unspecified percutaneous transluminal balloon angioplasty and insertion of stent into coronary artery   **Coronary artery bypass graft surgery (CABG)**  K40 Saphenous vein graft replacement of coronary artery   - K40.1 Saphenous vein graft replacement of one coronary artery - K40.2 Saphenous vein graft replacement of two coronary arteries - K40.3 Saphenous vein graft replacement of three coronary arteries - K40.4 Saphenous vein graft replacement of four or more coronary arteries - K40.8 Other specified saphenous vein graft replacement of coronary artery - K40.9 Unspecified saphenous vein graft replacement of coronary artery   K41 Other autograft replacement of coronary artery   - K41.1 Autograft replacement of one coronary artery NEC - K41.2 Autograft replacement of two coronary arteries NEC - K41.3 Autograft replacement of three coronary arteries NEC - K41.4 Autograft replacement of four or more coronary arteries NEC - K41.8 Other specified other autograft replacement of coronary artery - K41.9 Unspecified other autograft replacement of coronary artery   K42 Allograft replacement of coronary artery   - K42.1 Allograft replacement of one coronary artery - K42.2 Allograft replacement of two coronary arteries - K42.3 Allograft replacement of three coronary arteries - K42.4 Allograft replacement of four or more coronary arteries - K42.8 Other specified allograft replacement of coronary artery - K42.9 Unspecified allograft replacement of coronary artery   K43 Prosthetic replacement of coronary artery   - K43.1 Prosthetic replacement of one coronary artery - K43.2 Prosthetic replacement of two coronary arteries - K43.3 Prosthetic replacement of three coronary arteries - K43.4 Prosthetic replacement of four or more coronary arteries - K43.8 Other specified prosthetic replacement of coronary artery - K43.9 Unspecified prosthetic replacement of coronary artery   K44 Other replacement of coronary artery-   - K44.1 Replacement of coronary arteries using multiple methods - K44.2 Revision of replacement of coronary artery - K44.8 Other specified other replacement of coronary artery - K44.9 Unspecified other replacement of coronary artery   K45 Connection of thoracic artery to coronary artery   - K45.1 Double anastomosis of mammary arteries to coronary arteries - K45.2 Double anastomosis of thoracic arteries to coronary arteries NEC - K45.3 Anastomosis of mammary artery to left anterior descending coronary artery - K45.4 Anastomosis of mammary artery to coronary artery NEC - K45.5 Anastomosis of thoracic artery to coronary artery NEC - K45.6 Revision of connection of thoracic artery to coronary artery - K45.8 Other specified connection of thoracic artery to coronary artery - K45.9 Unspecified connection of thoracic artery to coronary artery   K46 Other bypass of coronary artery   - K46.1 Double implantation of mammary arteries into heart - K46.2 Double implantation of thoracic arteries into heart NEC - K46.3 Implantation of mammary artery into heart NEC - K46.4 Implantation of thoracic artery into heart NEC - K46.5 Revision of implantation of thoracic artery into heart - K46.8 Other specified other bypass of coronary artery - K46.9 Unspecified other bypass of coronary artery |
| CVD death  (ICD-10) | Cause of death (CVD) using the ICD-10 codes: I0, I11, I13, I20-I79 |

**Supplementary Table S3**. Baseline characteristics of the UK Biobank participants by inclusion status in the analytical sample.

|  | **Included** | **Excluded** |
| --- | --- | --- |
| n | 3,612 | 2,358 |
| MEDLIFE | 8.72 (2.48) | 8.78 (2.54) |
| Age, years | 59.8 (6.61) | 57.5 (7.61) |
| Sex, female (%) | 1,222 (33.8) | 1,074 (45.6) |
| Ethnicity, n (%) |  |  |
| - White | 3,375 (93.4) | 2,156 (92.6) |
| - Non-white | 237 (6.6) | 173 (7.4) |
| Education, n (%) |  |  |
| - University education | 1,330 (36.8) | 925 (39.8) |
| - Non-university education | 2,282 (63.2) | 1,402 (60.3) |
| Deprivation index | -1.22 (3.04) | -1.10 (3.02) |
| Family history of CVD, n (%) | 2,379 (65.9) | 1,371 (61.6) |
| Region of assessment, n (%) |  |  |
| - England | 3,334 (92.3) | 2,120 (89.9) |
| - Wales | 130 (3.60) | 114 (4.83) |
| - Scotland | 148 (4.10) | 124 (5.26) |
| Smoking status, n (%) |  |  |
| - Never | 1,583 (43.8) | 1,115 (47.7) |
| - Former | 1,756 (48.6) | 1,001 (42.8) |
| - Current | 273 (7.56) | 223 (9.5) |
| Energy intake (Kcal/day) | 2043.2 (521.3) | 2058.4 (531.5) |
| Vitamin/Mineral supplement, n (%) | 1,691 (46.8) | 1,169 (49.6) |
| Body Mass Index (kg/m^2^)* , n (%) |  |  |
| - Normal/Overweight | 1,806 (50.0) | 1,220 (52.3) |
| - Obese | 1,806 (50.0) | 1,111 (47.7) |
| Hypertension, n (%) | 2,596 (71.9) | 1,350 (57.3) |
| Major adverse cardiovascular events, n (%) | 605 (16.8) | 388 (16.5) |
| Cancer, n (%) | 462 (12.8) | 343 (14.6) |
| Cardiovascular Disease Medications | 3.39 (1.82) | 2.12 (2.04) |
| HbA_1c_ (mmol/mol), n (%) |  |  |
| - <53 | 2,372 (65.7) | 1,278 (63.2) |
| - ≥53 | 1,240 (34.3) | 745 (36.9) |
| Age at diagnosis, n (%)* |  |  |
| - <55 | 1,734 (48.1) | 641 (68.9) |
| - ≥55 | 1,878 (52.0) | 290 (31.1) |
| Duration of Type 2 Diabetes, n (%)* |  |  |
| - <5.92 | 1,806 (50.0) | 1,562 (66.2) |
| - ≥5.92 | 1,806 (50.0) | 796 (33.8) |
| Type 2 Diabetes Medications, n (%) | 2,436 (67.4) | 860 (36.5) |

*Continuous variables are expressed as mean (standard deviation). Categorical variables are expressed as percentages (%). Abbreviations: MEDLIFE Mediterranean Lifestyle Index, CVD cardiovascular disease*

**Supplementary Table S4.** Hazard ratios (95% confidence interval) for major adverse cardiovascular events by MEDLIFE adherence (quartiles) according to subgroups of sociodemographic and clinical characteristics

| **MEDLIFE** | | | | | | |
| --- | --- | --- | --- | --- | --- | --- |
|  | Cases/n | Q1 | Q2 | Q3 | Q4 | P for interaction |
| **Sex** |  |  |  |  |  | 0.481 |
| Male | 573/2,390 | 1 Ref. | 1.02 (0.81, 1.28) | 0.79 (0.63, 0.98) | 0.74 (0.57, 0.95) |  |
| Female | 164/1,222 | 1 Ref. | 0.82 (0.57, 1.20) | 0.56 (0.32, 0.99) | 0.77 (0.49, 1.19) |  |
| **Ethnicity** |  |  |  |  |  | 0.957 |
| Non-White | 35/237 | 1 Ref. | 0.92 (0.31, 2.74) | 0.99 (0.35, 2.83) | 0.86 (0.30, 2.47) |  |
| White | 702/3,375 | 1 Ref. | 0.95 (0.78, 1.16) | 0.74 (0.60, 0.91) | 0.72 (0.58, 0.90) |  |
| **Education** |  |  |  |  |  | **0.025** |
| University | 241/1,330 | 1 Ref. | 0.96 (0.67, 1.38) | 0.49 (0.34, 0.73) | 0.83 (0.59, 1.17) |  |
| Non-university | 496/2,282 | 1 Ref. | 0.95 (0.75, 1.20) | 0.89 (0.70, 1.14) | 0.65 (0.49, 0.87) |  |
| **Deprivation Index*** |  |  |  |  |  | 0.551 |
| <-2.01 | 347/1,805 | 1 Ref. | 0.80 (0.59, 1.08) | 0.69 (0.52, 0.91) | 0.75 (0.55,1.03) |  |
| ≥-2.01 | 390/1,807 | 1 Ref. | 1.08 (0.83, 1.39) | 0.77 (0.57, 1.04) | 0.73 (0.54, 0.99) |  |
| **Family History of CVD** |  |  |  |  |  | 0.595 |
| Yes | 546/2,379 | 1 Ref. | 0.95 (0.75, 1.19) | 0.81 (0.64, 1.03) | 0.74 (0.58, 0.95) |  |
| No | 191/1,233 | 1 Ref. | 0.93 (0.64, 1.37) | 0.55 (0.36, 0.84) | 0.66 (0.43, 1.02) |  |
| **Smoking Status** |  |  |  |  |  | 0.194 |
| Never | 257/1,583 | 1 Ref. | 1.03 (0.74, 1.42) | 0.53 (0.36, 0.76) | 0.74 (0.52, 1.06) |  |
| Former | 413/1,756 | 1 Ref. | 0.82 (0.62, 1.08) | 0.84 (0.65, 1.08) | 0.69 (0.52, 0.93) |  |
| Current | 67/273 | 1 Ref. | 1.16 (0.62, 2.19) | 0.90 (0.38, 2.14) | 0.68 (0.25, 1.85) |  |
| **Vitamin/Mineral Use** |  |  |  |  |  | 0.649 |
| Yes | 359/1,691 | 1 Ref. | 0.98 (0.74, 1.29) | 0.79 (0.59, 1.05) | 0.70 (0.51, 0.96) |  |
| No | 378/1,921 | 1 Ref. | 0.93 (0.70, 1.22) | 0.71 (0.54, 0.95) | 0.78 (0.57, 1.05) |  |
| **Body Mass Index*** |  |  |  |  |  | 0.716 |
| <30.3 | 326/1,806 | 1 Ref. | 0.83 (0.61, 1.14) | 0.70 (0.52, 0.95) | 0.71 (0.51, 0.97) |  |
| ≥30.3 | 411/1,806 | 1 Ref. | 1.01 (0.78, 1.30) | 0.75 (0.56, 0.99) | 0.75 (0.56, 1.01) |  |
| **Hypertension** |  |  |  |  |  | 0.977 |
| Yes | 680/3,051 | 1 Ref. | 0.94 (0.77, 1.16) | 0.74 (0.60, 0.91) | 0.74 (0.59, 0.92) |  |
| No | 57/561 | 1 Ref. | 0.80 (0.39, 1.66) | 0.64 (0.29, 1.43) | 0.71 (0.32, 1.55) |  |
| **MACE** |  |  |  |  |  | 0.285 |
| Yes | 317/605 | 1 Ref. | 1.15 (0.85, 1.55) | 0.90 (0.66, 1.22) | 0.75 (0.52, 1.07) |  |
| No | 420/3,007 | 1 Ref. | 0.85 (0.65, 1.10) | 0.66 (0.50, 0.87) | 0.76 (0.58, 0.99) |  |
| **Cancer** |  |  |  |  |  | 0.503 |
| Yes | 105/462 | 1 Ref. | 1.00 (0.57, 1.74) | 0.99 (0.59, 1.67) | 0.67 (0.37, 1.21) |  |
| No | 632/3,150 | 1 Ref. | 0.94 (0.76, 1.16) | 0.71 (0.57, 0.89) | 0.73 (0.58, 0.92) |  |
| **Medications for CVD*** |  |  |  |  |  | 0.423 |
| <2 | 131/1,058 | 1 Ref. | 0.82 (0.51, 1.32) | 0.62 (0.38, 1.01) | 0.48 (0.29, 0.79) |  |
| ≥2 | 606/2,554 | 1 Ref. | 0.98 (0.79, 1.22) | 0.78 (0.62, 0.97) | 0.78 (0.61, 0.99) |  |
| **HbA_1c (mmol/)_** |  |  |  |  |  | 0.149 |
| <53 | 458/2,372 | 1 Ref. | 1.12 (0.87, 1.44) | 0.76 (0.58, 0.99) | 0.84 (0.64, 1.09) |  |
| ≥53 | 279/1,240 | 1 Ref. | 0.73 (0.53, 1.01) | 0.75 (0.54, 1.04) | 0.57 (0.38, 0.84) |  |
| **Age at diagnosis*** |  |  |  |  |  | 0.128 |
| <55 | 322/1,734 | 1 Ref. | 1.00 (0.75, 1.34) | 0.74 (0.54, 1.01) | 0.54 (0.38, 0.78) |  |
| ≥55 | 415/1,878 | 1 Ref. | 0.94 (0.72, 1.22) | 0.76 (0.58, 0.99) | 0.90 (0.68, 1.19) |  |
| **Duration of Type 2 Diabetes*** |  |  |  |  |  | 0.586 |
| <5.92 | 341/1,806 | 1 Ref. | 1.01 (0.76, 1.34) | 0.70 (0.52, 0.96) | 0.77 (0.56, 1.06) |  |
| ≥5.92 | 396/1,806 | 1 Ref. | 0.88 (0.66, 1.16) | 0.77 (0.59, 1.01) | 0.71 (0.53, 0.95) |  |
| **Type 2 Diabetes Medications** |  |  |  |  |  | **0.049** |
| Yes | 521/2,436 | 1 Ref. | 0.81 (0.65, 1.03) | 0.73 (0.57, 0.93) | 0.63 (0.48, 0.82) |  |
| No | 216/1,176 | 1 Ref. | 1.56 (1.07, 2.28) | 0.84 (0.57, 1.24) | 1.07 (0.71, 1.60) |  |

*Values of all models are hazard ratios (95% confidence interval)*

*Abbreviations: MEDLIFE Mediterranean Lifestyle Index, Q quartiles, CVD Cardiovascular Disease*

**Medians*

Adjusted for sex, ethnicity (white, non-white), education (university, non-university education), deprivation index, family history of cardiovascular disease, region of assessment, smoking status (never, past, and current smoker), energy intake (Kcal), vitamin/mineral supplement (yes/no), Body Mass Index (under, equal or over the median), hypertension (yes/no), prevalent MACE (yes/no), cancer (yes/no), number of medications for CVD, HbA_1c_ (<53 or ≥53 mmol/mol), age at T2D diagnosis (under, equal or over the median), duration of diabetes (under, equal or over the median), and medications for T2D (yes/no)

**Supplementary Table S5.** Hazard Ratios (95% confidence interval) for major adverse cardiovascular events, cardiovascular and all-cause mortality by MEDLIFE adherence (quartiles) in participants with ≥three dietary assessments

| MEDLIFE | | | | | | |
| --- | --- | --- | --- | --- | --- | --- |
|  | Q1 (lowest) | Q2 | Q3 | Q4 (highest) | p-trend | Per 2-point increment |
| MEDLIFE | 5.97 (1.18) | 8.25 (0.43) | 9.56 (0.49) | 12.0 (1.26) |  |  |
| **Major Adverse Cardiovascular Events** | | | | | | |
| Cases/N | 180/809 | 100/480 | 86/458 | 66/414 |  | 432/2,161 |
| Person-years | 7,300.2 | 4,371.7 | 4,208.5 | 3,945.1 |  | 19,825.4 |
| Model 1^a^ | 1 Ref. | 1.02 (0.80, 1.31) | 0.74 (0.57, 0.96) | 0.67 (0.51, 0.89) | p<0.01 | 0.85 (0.79, 0.93) |
| Model 2^b^ | 1 Ref. | 1.00 (0.78, 1.29) | 0.74 (0.57, 0.97) | 0.66 (0.50, 0.89) | p<0.01 | 0.85 (0.78, 0.93) |
| Model 3^c^ | 1 Ref. | 0.97 (0.76, 1.25) | 0.73 (0.56, 0.95) | 0.73 (0.55, 0.98) | p<0.01 | 0.87 (0.80, 0.95) |
| Model 4^d^ | 1 Ref. | 1.00 (0.77, 1.28) | 0.74 (0.57, 0.96) | 0.75 (0.56, 1.01) | p=0.011 | 0.89 (0.81, 0.97) |
| **All-cause Mortality** | | | | | | |
| Cases/N | 168/809 | 94/480 | 80/458 | 60/414 |  | 402/2,161 |
| Person-years | 8,940.9 | 5,370.3 | 5,140.7 | 4,762.4 |  | 24,214.4 |
| Model 1^a^ | 1 Ref. | 0.96 (0.75, 1.25) | 0.74 (0.56, 0.96) | 0.65 (0.48, 0.88) | p<0.01 | 0.86 (0.79, 0.94) |
| Model 2^b^ | 1 Ref. | 0.98 (0.75, 1.26) | 0.78 (0.59, 1.02) | 0.69 (0.51, 0.94) | p<0.01 | 0.88 (0.81, 0.96) |
| Model 3^c^ | 1 Ref. | 0.97 (0.75, 1.26) | 0.77 (0.58, 1.01) | 0.71 (0.53, 0.97) | p=0.01 | 0.89 (0.82, 0.97) |
| Model 4^d^ | 1 Ref. | 0.97 (0.75, 1.26) | 0.77 (0.58, 1.01) | 0.72 (0.53, 0.98) | p=0.012 | 0.90 (0.82, 0.98) |
| **Cardiovascular Mortality** | | | | | | |
| Cases/N | 48/809 | 34/480 | 18/458 | 10/414 |  | 110/2,161 |
| Person-years | 8,940.9 | 5,370.3 | 5,140.7 | 4,762.4 |  | 24,214.4 |
| Model 1^a^ | 1 Ref. | 1.38 (0.88, 2.16) | 0.58 (0.33, 0.99) | 0.38 (0.19, 0.76) | p<0.01 | 0.73 (0.62, 0.86) |
| Model 2^b^ | 1 Ref. | 1.34 (0.85, 2.11) | 0.59 (0.34, 1.03) | 0.38 (0.19, 0.77) | p<0.01 | 0.74 (0.62, 0.87) |
| Model 3^c^ | 1 Ref. | 1.29 (0.82, 2.03) | 0.57 (0.33, 0.99) | 0.42 (0.21, 0.83) | p<0.01 | 0.75 (0.64, 0.89) |
| Model 4^d^ | 1 Ref. | 1.30 (0.82, 2.05) | 0.56 (0.32, 0.98) | 0.42 (0.21, 0.85) | p<0.01 | 0.76 (0.64, 0.90) |

*Values of all models are hazard ratios (95% confidence interval)*

*Abbreviations: MEDLIFE Mediterranean Lifestyle Index, Q quartiles,*

^a^ Adjusted for sex, ethnicity (white, non-white), education (university, non-university education), deprivation index, family history of cardiovascular disease (yes/no), and region of assessment.

^b^ Adjusted as for Model 1 + smoking status (never, past, and current smoker), energy intake (Kcal), vitamin/mineral supplement (yes/no) and Body Mass Index (under/ equal or over the median)

^c^ Adjusted as for Model 2 + hypertension (yes/no), prevalent MACE (yes/no), cancer (yes/no), and number of medications for CVD.

^d^ Adjusted as for Model 3 + HbA_1c_ (<53 or ≥53 mmol/mol), age at T2D diagnoses (under, equal or over the median), duration of diabetes (under, equal or over the median), and medications for T2D (yes/no).

**Supplementary Table S6.** Hazard Ratios (95% confidence interval) for major adverse cardiovascular events, cardiovascular and all-cause mortality by MEDLIFE adherence excluding participants who suffered a MACE during the initial two years of follow-up

| MEDLIFE | | | | | | |
| --- | --- | --- | --- | --- | --- | --- |
|  | Q1 (lowest) | Q2 | Q3 | Q4 (highest) | p-trend | Per 2-point increment |
| MEDLIFE | 6.00 (1.14) | 8.26 (0.44) | 9.58 (0.49) | 12.0 (1.29) |  |  |
| **Major Adverse Cardiovascular Events** | | | | | | |
| Cases/N | 242/1,190 | 137/748 | 128/771 | 109/740 |  | 616/3,449 |
| Person-years | 11,338.9 | 7,313.4 | 7,424.4 | 7,338.6 |  | 33,415.2 |
| Model 1^a^ | 1 Ref. | 0.93 (0.75, 1.15) | 0.70 (0.57, 0.87) | 0.66 (0.53, 0.83) | p<0.001 | 0.84 (0.79, 0.90) |
| Model 2^b^ | 1 Ref. | 0.94 (0.76, 1.16) | 0.73 (0.59, 0.91) | 0.69 (0.54, 0.87) | p<0.001 | 0.86 (0.80, 0.92) |
| Model 3^c^ | 1 Ref. | 0.91 (0.74, 1.13) | 0.72 (0.58, 0.89) | 0.72 (0.57, 0.90) | p<0.001 | 0.87 (0.81, 0.93) |
| Model 4^d^ | 1 Ref. | 0.92 (0.75, 1.14) | 0.74 (0.59, 0.92) | 0.73 (0.58, 0.92) | p<0.01 | 0.87 (0.82, 0.94) |
| **All-cause Mortality** | | | | | | |
| Cases/N | 212/1,190 | 115/748 | 129/771 | 92/740 |  | 548/3,449 |
| Person-years | 13,654.1 | 8,715.9 | 8,845.2 | 8,715.5 |  | 39,930.9 |
| Model 1^a^ | 1 Ref. | 0.87 (0.69, 1.10) | 0.81 (0.65, 1.01) | 0.63 (0.49, 0.81) | p<0.001 | 0.87 (0.81, 0.93) |
| Model 2^b^ | 1 Ref. | 0.90 (0.71, 1.13) | 0.87 (0.70, 1.09) | 0.69 (0.54, 0.89) | p<0.01 | 0.90 (0.83, 0.97) |
| Model 3^c^ | 1 Ref. | 0.90 (0.71, 1.13) | 0.87 (0.69, 1.08) | 0.71 (0.55, 0.91) | p<0.01 | 0.91 (0.84, 0.97) |
| Model 4^d^ | 1 Ref. | 0.91 (0.72, 1.15) | 0.88 (0.71, 1.11) | 0.73 (0.56, 0.93) | p=0.016 | 0.91 (0.85, 0.98) |
| **Cardiovascular Mortality** | | | | | | |
| Cases/N | 59/1,190 | 38/748 | 26/771 | 19/740 |  | 142/3,449 |
| Person-years | 13,654.1 | 8,715.9 | 8,845.2 | 8,715.5 |  | 39,930.9 |
| Model 1^a^ | 1 Ref. | 1.13 (0.75, 1.72) | 0.56 (0.35, 0.90) | 0.46 (0.28, 0.78) | p<0.001 | 0.77 (0.67, 0.89) |
| Model 2^b^ | 1 Ref. | 1.15 (0.76, 1.75) | 0.60 (0.37, 0.95) | 0.49 (0.29, 0.83) | p<0.01 | 0.79 (0.68, 0.91) |
| Model 3^c^ | 1 Ref. | 1.12 (0.74, 1.69) | 0.58 (0.36, 0.94) | 0.51 (0.30, 0.86) | p<0.01 | 0.80 (0.69, 0.92) |
| Model 4^d^ | 1 Ref. | 1.13 (0.74, 1.72) | 0.59 (0.37, 0.95) | 0.51 (0.30, 0.87) | p<0.01 | 0.80 (0.69, 0.93) |

*Values of all models are hazard ratios (95% confidence interval)*

*Abbreviations: MEDLIFE Mediterranean Lifestyle Index, Q quartiles,*

^a^ Adjusted for sex, ethnicity (white, non-white), education (university, non-university education), deprivation index, family history of cardiovascular disease (yes/no), and region of assessment.

^b^ Adjusted as for Model 1 + smoking status (never, past, and current smoker), energy intake (Kcal), vitamin/mineral supplement (yes/no) and Body Mass Index (under/ equal or over the median)

^c^ Adjusted as for Model 2 + hypertension (yes/no), prevalent MACE (yes/no), cancer (yes/no), and number of medications for CVD.

^d^ Adjusted as for Model 3 + HbA_1c_ (<53 or ≥53 mmol/mol), age at T2D diagnoses (under, equal or over the median), duration of diabetes (under, equal or over the median), and medications for T2D (yes/no).

**Supplementary Table S7.** Hazard ratios (95% confidence interval) for major adverse cardiovascular events, cardiovascular, and all-cause mortality by MEDLIFE adherence (quartiles) additionally adjusting for dyslipidemia

| MEDLIFE | | | | | | |
| --- | --- | --- | --- | --- | --- | --- |
|  | Q1 (lowest) | Q2 | Q3 | Q4 (highest) | p-trend | Per 2-point increment |
| MEDLIFE | 6.01 (1.14) | 8.25 (0.43) | 9.57 (0.49) | 12.0 (1.28) |  |  |
| **Major Adverse Cardiovascular Events** | | | | | | |
| Cases/N | 292/1,258 | 167/789 | 151/798 | 127/767 |  | 737/3,612 |
| Person-years | 11,409.5 | 7,360.1 | 7,456.4 | 7,365.7 |  | 33,591.6 |
| Model 1^a^ | 1 Ref. | 0.97 (0.80, 1.17) | 0.71 (0.58, 0.86) | 0.67 (0.54, 0.82) | p<0.001 | 0.85 (0.80, 0.91) |
| Model 2^b^ | 1 Ref. | 0.96 (0.79, 1.17) | 0.73 (0.60, 0.89) | 0.68 (0.55, 0.84) | p<0.001 | 0.86 (0.81, 0.91) |
| Model 3^c^ | 1 Ref. | 0.94 (0.77, 1.14) | 0.72 (0.59, 0.89) | 0.72 (0.58, 0.89) | p<0.001 | 0.87 (0.82, 0.93) |
| Model 4^d^ | 1 Ref. | 0.95 (0.78, 1.16) | 0.74 (0.61, 0.91) | 0.73 (0.59, 0.91) | p<0.001 | 0.88 (0.83, 0.94) |
| **All-cause Mortality** | | | | | | |
| Cases/N | 251/1,258 | 140/789 | 146/798 | 108/767 |  | 645/3,612 |
| Person-years | 14,102.5 | 9,000.0 | 9,033.9 | 8,895.0 |  | 41,031.5 |
| Model 1^a^ | 1 Ref. | 0.91 (0.74, 1.12) | 0.80 (0.65, 0.98) | 0.65 (0.52, 0.82) | p<0.001 | 0.87 (0.82, 0.93) |
| Model 2^b^ | 1 Ref. | 0.93 (0.75, 1.14) | 0.85 (0.69, 1.04) | 0.69 (0.55, 0.88) | p<0.01 | 0.90 (0.84, 0.96) |
| Model 3^c^ | 1 Ref. | 0.93 (0.75, 1.14) | 0.84 (0.68, 1.04) | 0.72 (0.57, 0.91) | p<0.01 | 0.91 (0.85, 0.97) |
| Model 4^d^ | 1 Ref. | 0.94 (0.76, 1.15) | 0.85 (0.69, 1.05) | 0.73 (0.58, 0.93) | p<0.01 | 0.91 (0.85, 0.98) |
| **Cardiovascular Mortality** | | | | | | |
| Cases/N | 70/1,258 | 48/789 | 32/798 | 21/767 |  | 171/3,612 |
| Person-years | 14,102.5 | 9,000.0 | 9,033.9 | 8,895.0 |  | 41,031.5 |
| Model 1^a^ | 1 Ref. | 1.21 (0.83, 1.75) | 0.60 (0.40, 0.92) | 0.44 (0.27, 0.73) | p<0.001 | 0.76 (0.67, 0.87) |
| Model 2^b^ | 1 Ref. | 1.21 (0.83, 1.76) | 0.63 (0.41, 0.96) | 0.46 (0.28, 0.75) | p<0.001 | 0.77 (0.68, 0.88) |
| Model 3^c^ | 1 Ref. | 1.18 (0.81, 1.71) | 0.62 (0.40, 0.95) | 0.48 (0.29, 0.79) | p<0.001 | 0.78 (0.68, 0.89) |
| Model 4^d^ | 1 Ref. | 1.20 (0.82, 1.75) | 0.62 (0.40, 0.95) | 0.49 (0.29, 0.80) | p<0.001 | 0.79 (0.69, 0.90) |

^a^ Adjusted for sex, ethnicity (white, non-white), education (university, non-university education), deprivation index, family history of cardiovascular disease (yes/no), and region of assessment.

^b^ Adjusted as for Model 1 + smoking status (never, past, and current smoker), energy intake (Kcal), vitamin/mineral supplement (yes/no) and Body Mass Index (under/ equal or over the median)

^c^ Adjusted as for Model 2 + hypertension (yes/no), dyslipidemia (yes/no), prevalent MACE (yes/no), cancer (yes/no), and number of medications for CVD.

^d^ Adjusted as for Model 3 + HbA_1c_ (<53 or ≥53 mmol/mol), age at T2D diagnoses (under, equal or over the median), duration of diabetes (under, equal or over the median), and medications for T2D (yes/no).

**Supplementary Table S8.** Hazard ratios (95% confidence interval) for major adverse cardiovascular events, cardiovascular, and all-cause mortality by MEDLIFE adherence (quartiles) using the Fine-Gray method.

| MEDLIFE | | | | | | |
| --- | --- | --- | --- | --- | --- | --- |
|  | Q1 (lowest) | Q2 | Q3 | Q4 (highest) | p-trend | Per 2-point increment |
| MEDLIFE | 6.01 (1.14) | 8.25 (0.43) | 9.57 (0.49) | 12.0 (1.28) |  |  |
| **Major Adverse Cardiovascular Events** | | | | | | |
| Cases/N | 229/1,258 | 139/789 | 116/798 | 99/767 |  | 583/3,612 |
| Person-years | 11,409.5 | 7,360.1 | 7,456.4 | 7,365.7 |  | 33,591.6 |
| Model 1^a^ | 1 Ref. | 1.07 (0.86, 1.32) | 0.72 (0.58, 0.90) | 0.70 (0.55, 0.88) | p<0.001 | 0.86 (0.80, 0.92) |
| Model 2^b^ | 1 Ref. | 1.04 (0.84, 1.30) | 0.73 (0.58, 0.91) | 0.69 (0.54, 0.88) | p<0.001 | 0.86 (0.80, 0.92) |
| Model 3^c^ | 1 Ref. | 1.03 (0.83, 1.28) | 0.73 (0.58, 0.91) | 0.73 (0.57, 0.93) | p<0.01 | 0.87 (0.80, 0.94) |
| Model 4^d^ | 1 Ref. | 1.04 (0.84, 1.30) | 0.73 (0.58, 0.92) | 0.74 (0.57, 0.94) | p<0.01 | 0.87 (0.81, 0.94) |
| **Cardiovascular Mortality** | | | | | | |
| Cases/N | 70/1,258 | 48/789 | 32/798 | 21/767 |  | 171/3,612 |
| Person-years | 14,102.5 | 9,000.0 | 9,033.9 | 8,895.0 |  | 41,031.5 |
| Model 1^a^ | 1 Ref. | 1.25 (0.87, 1.81) | 0.62 (0.41, 0.95) | 0.47 (0.29, 0.77) | p<0.001 | 0.78 (0.68, 0.89) |
| Model 2^b^ | 1 Ref. | 1.25 (0.86, 1.82) | 0.64 (0.42, 0.98) | 0.48 (0.29, 0.80) | p<0.001 | 0.78 (0.68, 0.90) |
| Model 3^c^ | 1 Ref. | 1.21 (0.83, 1.76) | 0.63 (0.41, 0.96) | 0.50 (0.30, 0.84) | p<0.01 | 0.79 (0.68, 0.91) |
| Model 4^d^ | 1 Ref. | 1.23 (0.85, 1.79) | 0.62 (0.40, 0.96) | 0.51 (0.31, 0.85) | p<0.01 | 0.79 (0.68, 0.91) |

*Values of all models are hazard ratios (95% confidence interval)*

*Abbreviations: MEDLIFE Mediterranean Lifestyle Index, Q quartiles,*

^a^ Adjusted for sex, ethnicity (white, non-white), education (university, non-university education), deprivation index, family history of cardiovascular disease (yes/no), and region of assessment.

^b^ Adjusted as for Model 1 + smoking status (never, past, and current smoker), energy intake (Kcal), vitamin/mineral supplement (yes/no) and Body Mass Index (under/ equal or over the median)

^c^ Adjusted as for Model 2 + hypertension (yes/no), prevalent MACE (yes/no), cancer (yes/no), and number of medications for CVD.

^d^ Adjusted as for Model 3 + HbA_1c_ (<53 or ≥53 mmol/mol), age at T2D diagnoses (under, equal or over the median), duration of diabetes (under, equal or over the median), and medications for T2D (yes/no).

**Figure S1.** Directed Acyclic Graph of the total effects of the MEDLIFE on MACE

Nodes followed a left-to-right arrangement reflecting approximate chronology. The MEDLIFE is the exposure and MACE is the outcome. Pink nodes are ancestors of the exposure and outcome (confounders), and minimal sufficient adjustment set was considered to include for sex, ethnicity, education, deprivation index, family history of cardiovascular disease, smoking status, energy intake (Kcal), vitamin or mineral supplement, Body Mass Index, hypertension, prevalent major adverse cardiovascular events, cancer, number of medications for cardiovascular disease, HbA1c control, age at type 2 diagnosis, duration of diabetes, and medications for type 2 diabetes.

**Figure S2.** Association of the MEDLIFE with the individual major adverse cardiovascular events

Adjusted for sex, ethnicity (white, non-white), education (university, non-university education), deprivation index, family history of cardiovascular disease, region of assessment, smoking status (never, past, and current smoker), energy intake (Kcal), vitamin/mineral supplement (yes/no), Body Mass Index (under, equal or over the median), hypertension (yes/no), prevalent MACE (yes/no), cancer (yes/no), number of medications for CVD, HbA_1c_ (<53 or ≥53 mmol/mol), age at T2D diagnosis (under, equal or over the median), duration of diabetes (under, equal or over the median), and medications for T2D (yes/no).

*Abbreviations: MEDLIFE Mediterranean Lifestyle Index, CI Confidence Interval*

**Figure S3.** Restricted cubic spline regression for association of the MEDLIFE with risk of major adverse cardiovascular events (MACE), cardiovascular, and all-cause mortality excluding participants with a previous MACE (N=3,003).

Adjusted for sex, ethnicity (white, non-white), education (university, non-university education), deprivation index, family history of cardiovascular disease, region of assessment, smoking status (never, past, and current smoker), energy intake (Kcal), vitamin/mineral supplement (yes/no), Body Mass Index (under, equal or over the median), hypertension (yes/no), cancer (yes/no), number of medications for CVD, HbA_1c_ (<53 or ≥53 mmol/mol), age at T2D diagnosis (under, equal or over the median), duration of diabetes (under, equal or over the median), and medications for T2D (yes/no)

*Abbreviations: MEDLIFE Mediterranean Lifestyle Index, CI Confidence Interval.*

*The 3 knots were set at the 10^th^, 50^th^ and 90^th^ percentiles (6, 8 and 12 points, respectively). The histogram displays the frequency of scores within the MEDLIFE range (0–25). p for non-linearity>0.05*
